# Supplementary material for: Chromosome-level genome assembly and population genomics of Robinia pseudoacacia reveal the genetic basis for its wide cultivation
Source: Commun Biol. 2023 Jul 31;6:797. doi: 10.1038/s42003-023-05158-6 (PMC10390555; doi:10.1038/s42003-023-05158-6)
Supplement: Supplementary file 2 — Supplementary Files [file 42003_2023_5158_MOESM2_ESM.pdf]

Chromosome-level genome assembly and population genomics of *Robinia pseudoacacia* reveal the genetic basis for its wide cultivation

Zefu Wang[1,2,3#], Xiao Zhang[2#*], Hui Zhu[1], Weixiao Lei[1], Shengdan Wu[1*], Bingbing Liu[4*], Dafu Ru[1*]

[1] State Key Laboratory of Herbage Improvement and Grassland Agro-Ecosystem, College of Ecology, Lanzhou University, Lanzhou, 730000, China

[2] Tianjin Key Laboratory of Conservation and Utilization of Animal Diversity, College of Life Sciences, Tianjin Normal University, Tianjin, China

[3] Co-Innovation Center for Sustainable Forestry in Southern China, College of Biology and the Environment, Nanjing Forestry University, Nanjing, 210037, China

[4] Institute of Loess Plateau, Shanxi University, Taiyuan, 030006, China.

[#] Authors contributed equally.

[*] Author for correspondence. E-mail: rudf@lzu.edu.cn; zhangxiao@tjnu.edu.cn; lbb2015@sxu.edu.cn; wusd@lzu.edu.cn

Supplementary Figures

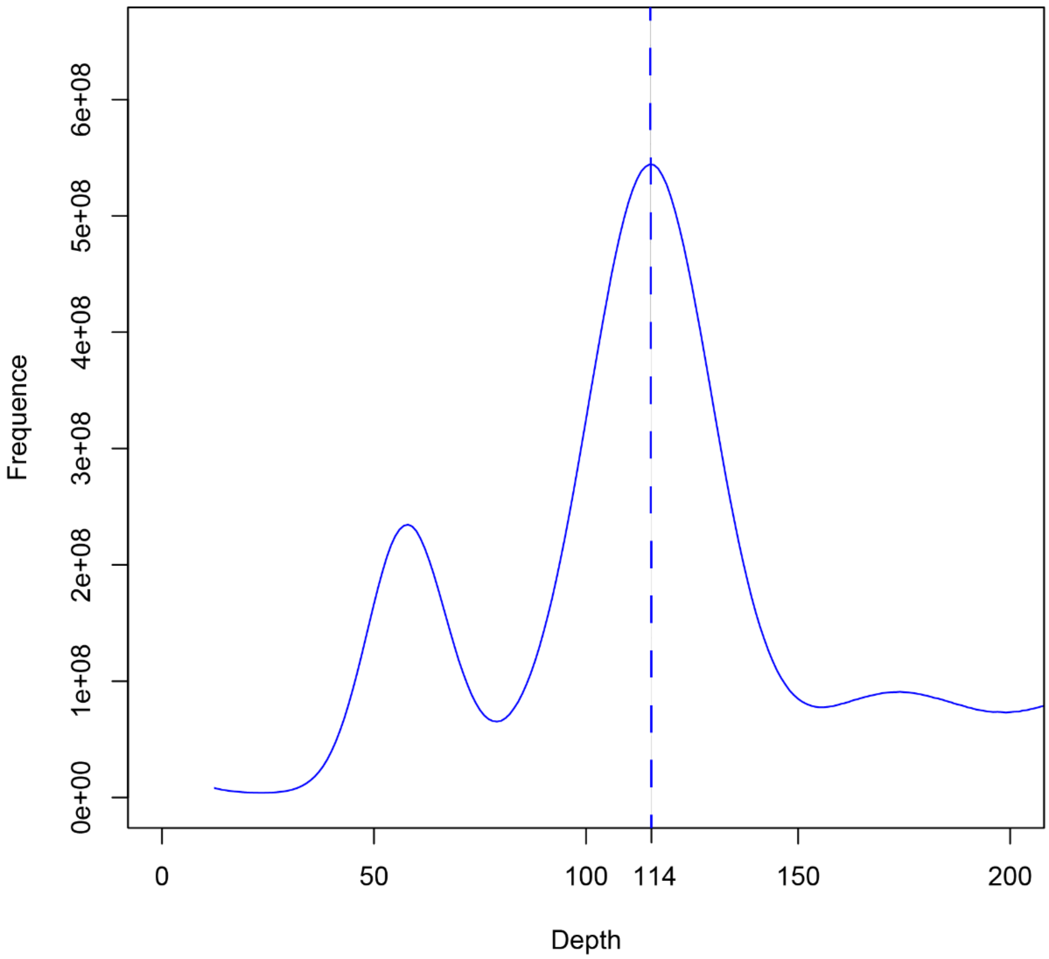

Supplementary Figure 1. Distribution of 17-mer frequency in the Illumina paired-end reads.

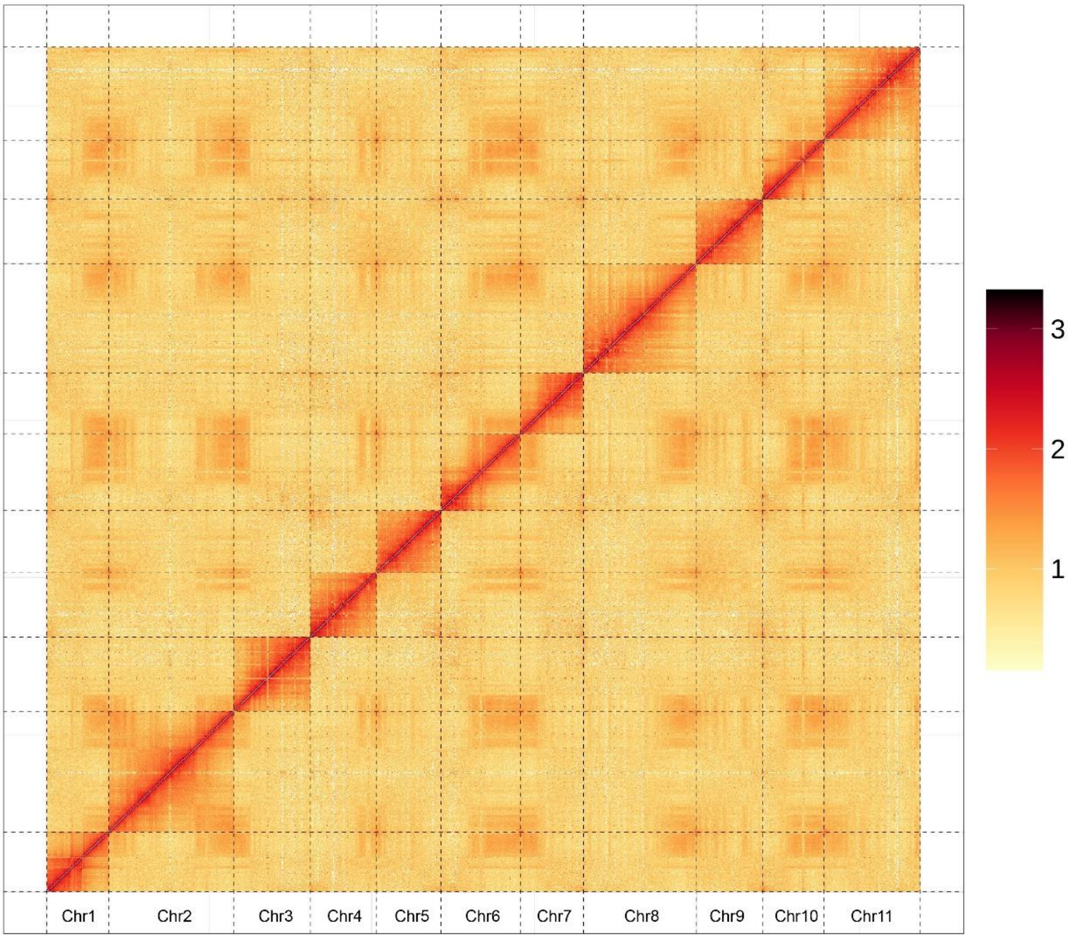

Supplementary Figure 2. Heat map of chromatin contact matrices generated by aligning a Hi-C dataset to the *R. pseudoacacia* genome.

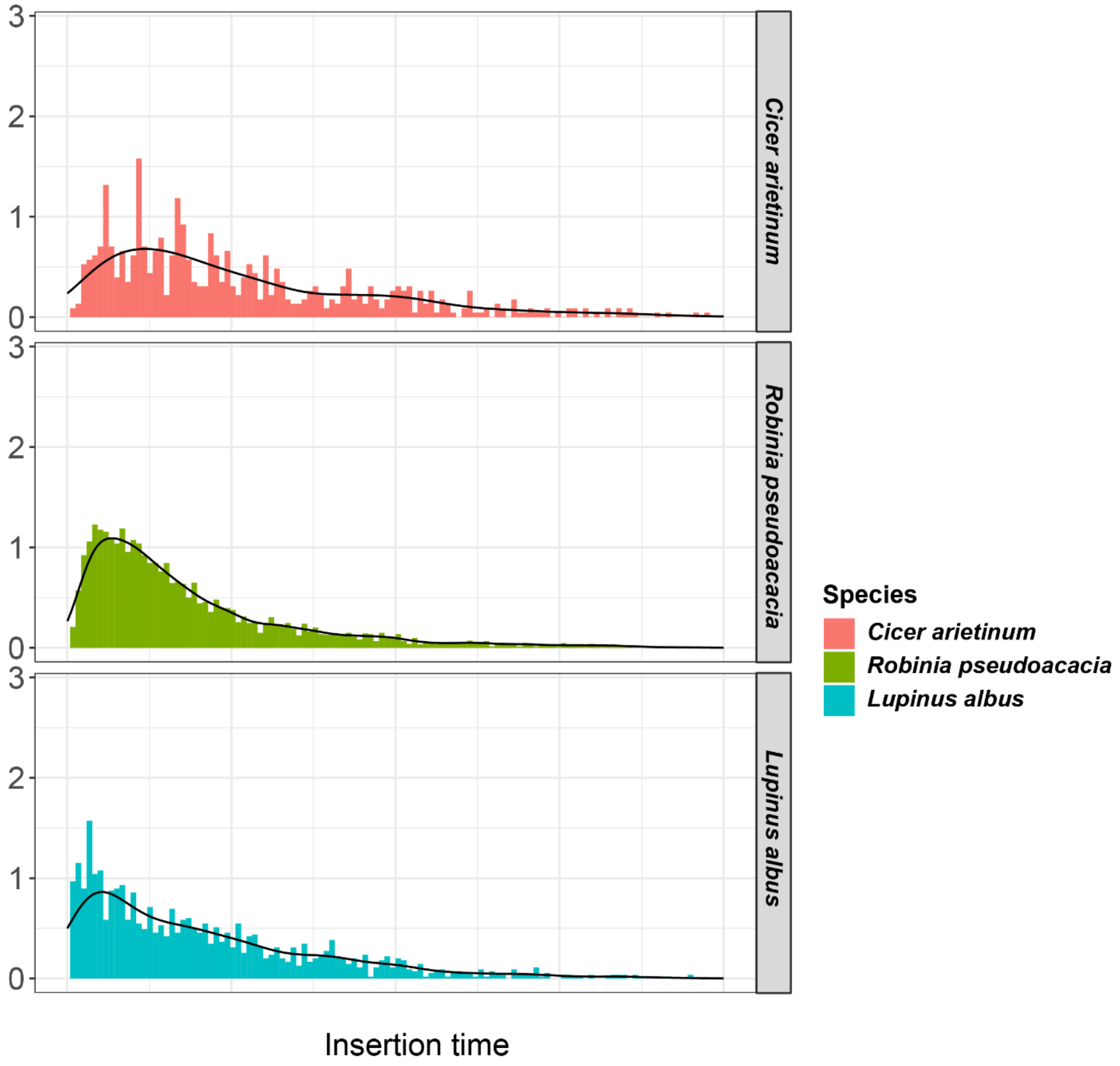

Supplementary Figure 3. Estimated insertion time of long terminal repeat retrotransposons (LTRs) in *Robinia pseudoacacia* and two other related species. The x-axis represents insertion time of the LTR, and the time was calculated using a substitution rate of 1.05e-8 substitutions per site per year.

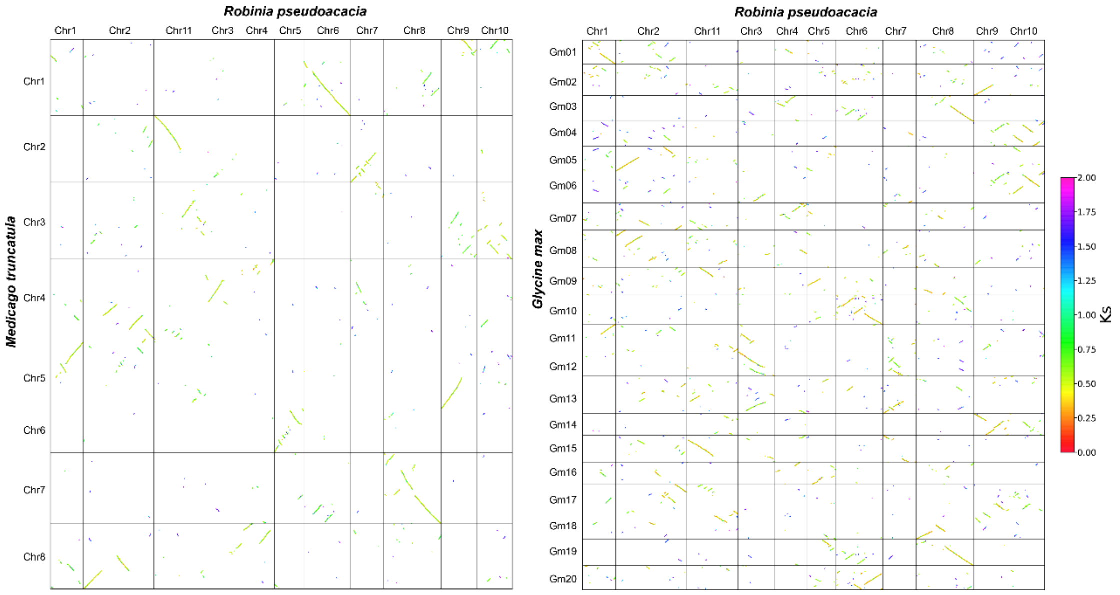

Supplementary Figure 4. Dot plot identified by wgdi between *R. pseudoacacia* and *M. truncatula* (left) / *G. max* (right).

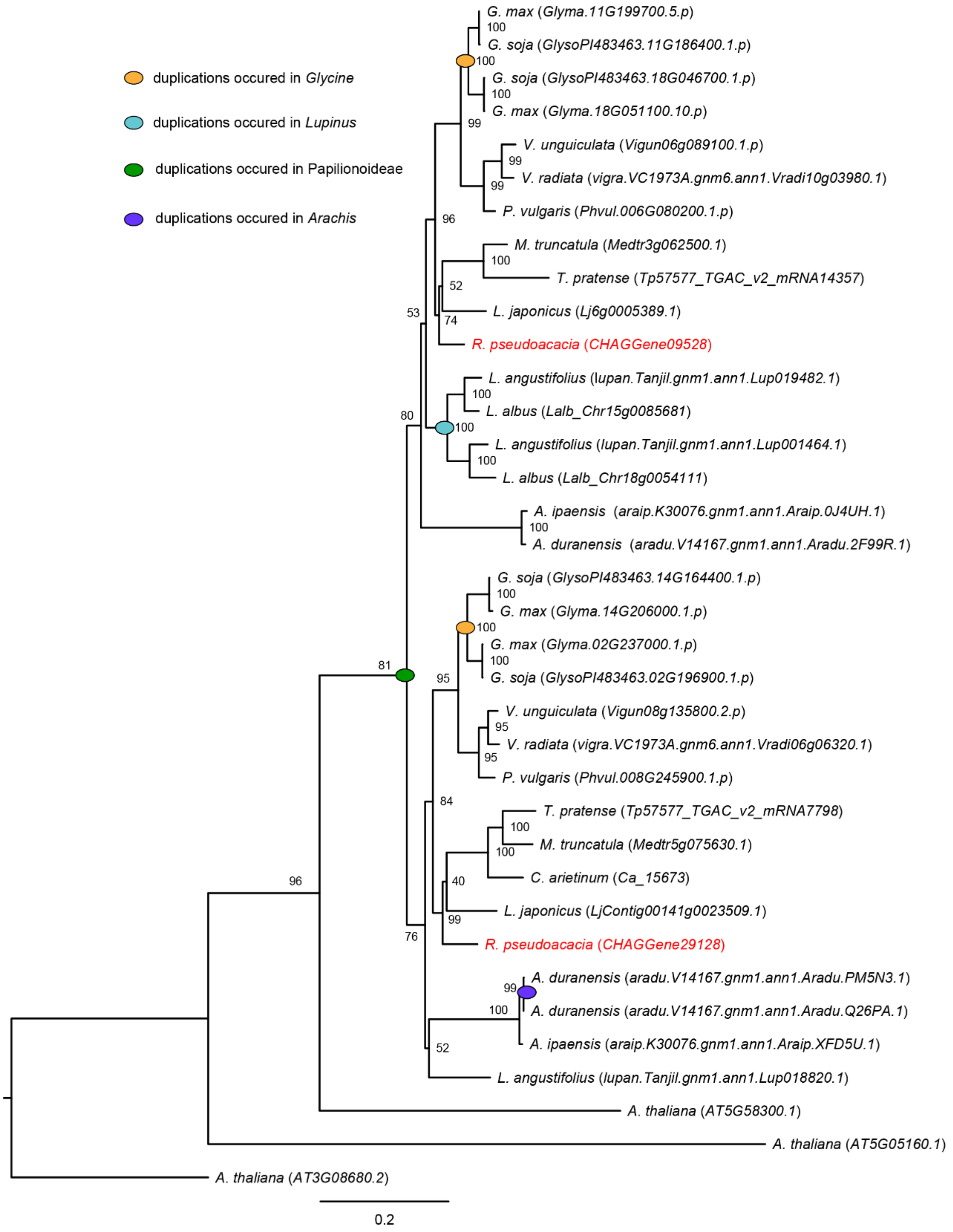

Supplementary Figure 5. Phylogeny of the OG0003003 gene family inferred using OrthoFinder showed duplications in its evolutionary history. Solid circles indicate the duplications that occurred in different periods. Numbers on branches show bootstrap values.

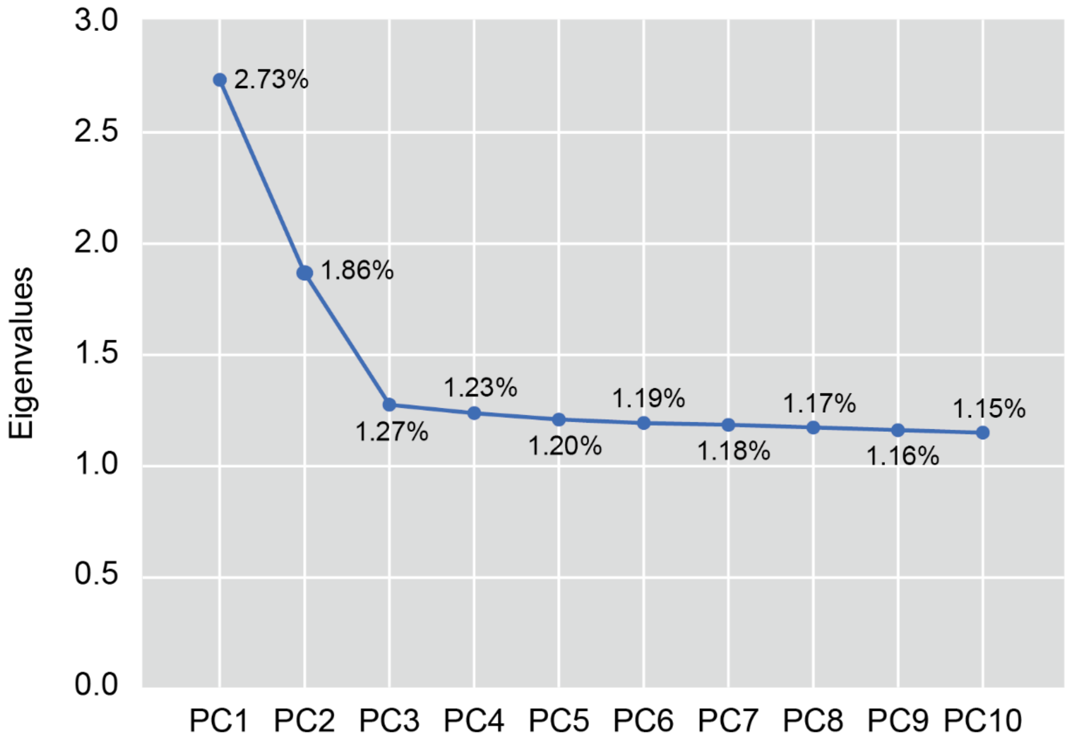

Supplementary Figure 6. PCs from 1-10 in PCA analysis.

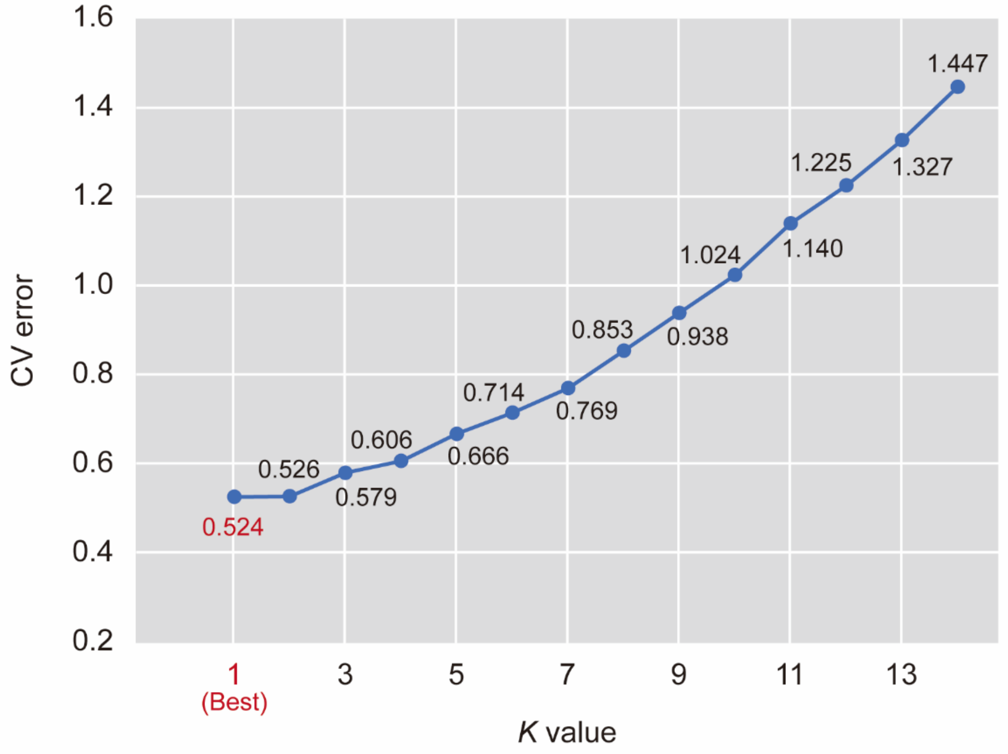

Supplementary Figure 7. Cross-validation error rates for different *K* values in Admixture analysis.

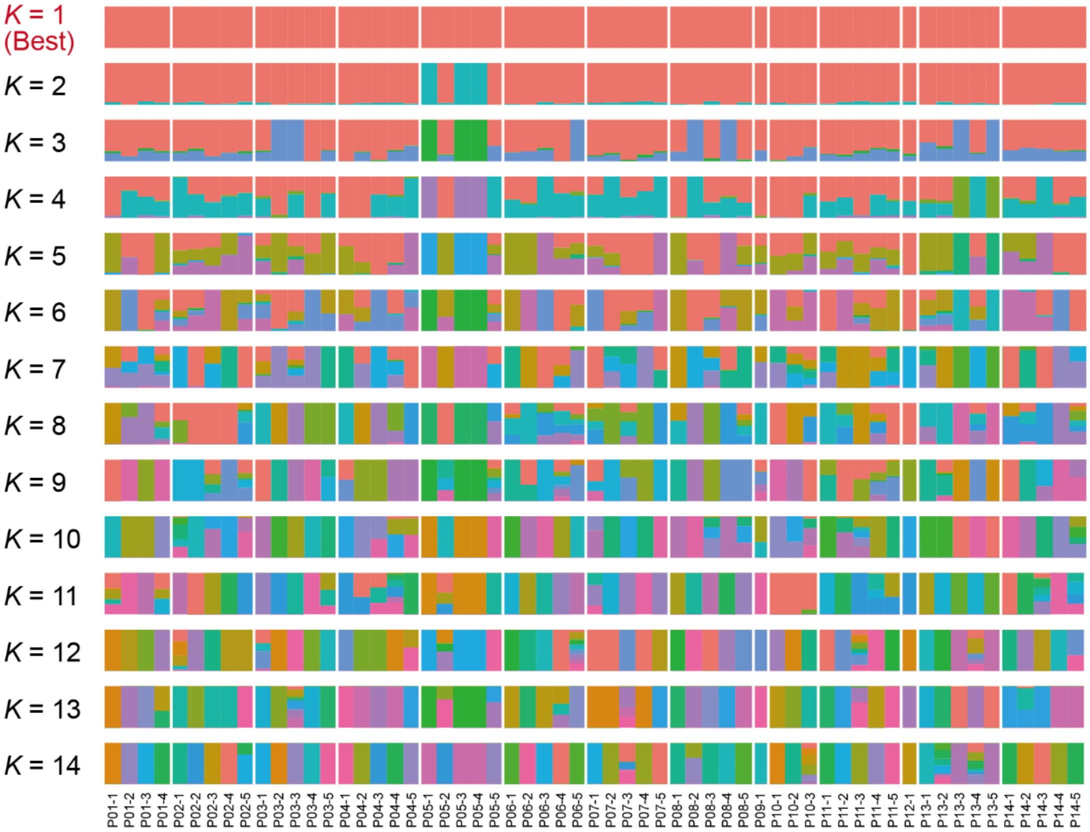

Supplementary Figure 8. Admixture proportions of genetic clusters for all individual of *R. pseudoacacia*. The scenarios of $K = 1$ to $K = 14$ are shown, and $K = 1$ is the best value according to cross-validation analysis.

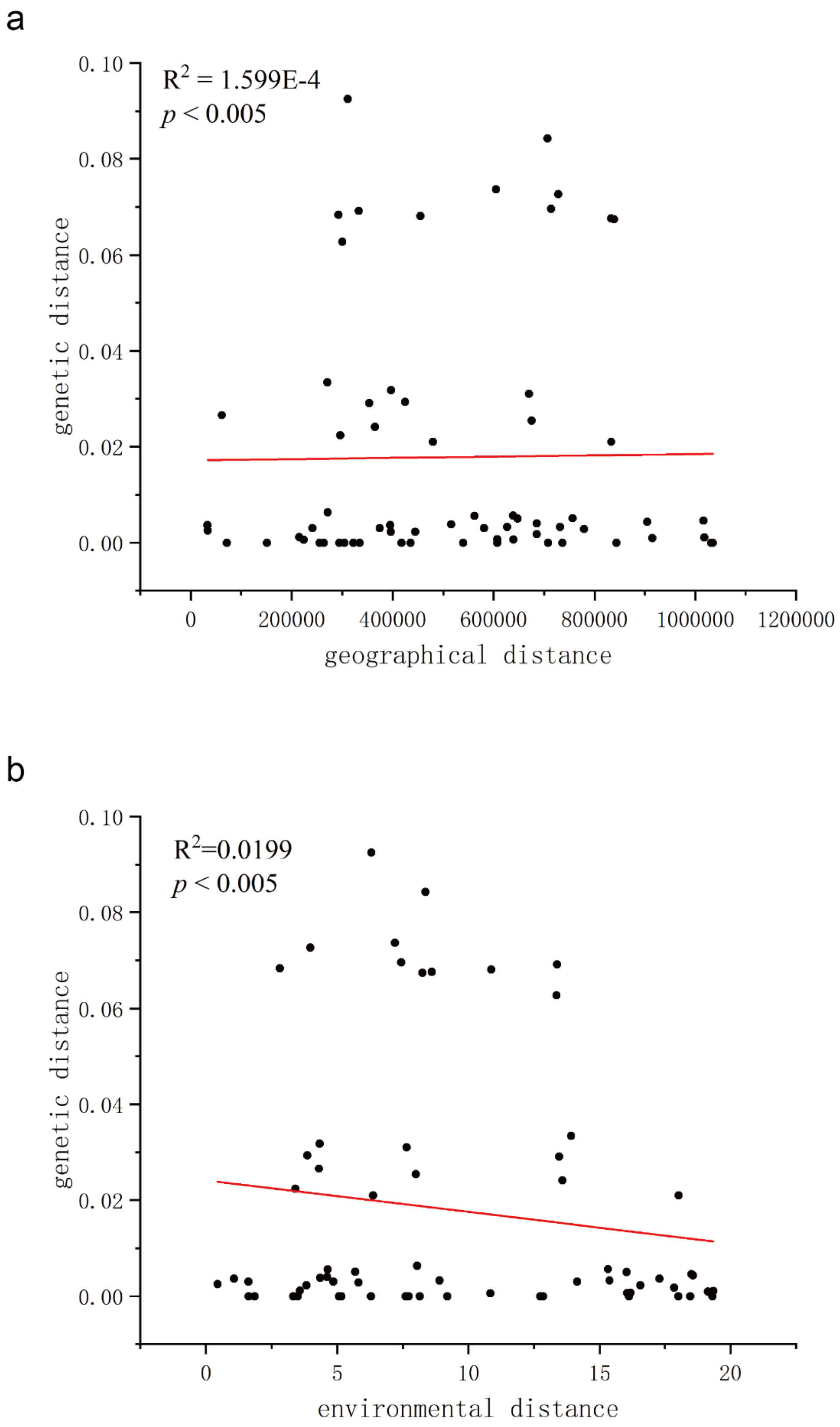

Supplementary Figure 9. Effects of geographical and environmental variables on genetic structure of *R. pseudoacacia* population. The relationship of genetic distance with (a) geographical distance and (b) environmental distance.

Supplementary Tables

Supplementary Table 1. Summary of sequencing data generated in this study

Library type	Read length	Total data(G)	Depth[a]	Application
Nanopore	31,726 bp (N50)	75.61	109.96×	Genome assembly
Illumina paired-end	2 × 150 bp	111.34	161.93×	Genome survey and base-level correction
Hi-C	2 × 150 bp	78.27	113.83×	Chromosome construction
RNA-Seq	2 × 150 bp	7.56 (flower)<br>6.32 (stem)<br>6.80 (leaf)	—	Genome annotation

[a]Depth was calculated according to an estimated genome size of 687.6 Mb

Supplementary Table 2. Summary of *R. pseudoacacia* genome assembly

Type	*R. pseudoacacia* assembly
Assembly size (bp)	682,403,908
Number of scaffolds	20
Max. scaffold length (bp)	96,078,175
Scaffold N50 size (bp)	59,867,354
Scaffold N90 size (bp)	48,438,208
Number of contigs	55
Max. contig length (bp)	49,345,383
Contig N50 size (bp)	32,134,000
Contig N90 size (bp)	11,176,400
GC content	33.33%

Supplementary Table 3. Estimation of *R. pseudoacacia* genome size based on the 17-mer method

K-mer	K-mer number	K-mer depth	Genome size	Revised genome size	Repeat rate	Heterozygosity rate
17	79,013,458,592	114	693.1 Mb	687.6 Mb	58.82%	1.13%

Supplementary Table 4. Summary of chromosome groups in the genome of *R. pseudoacacia* inferred using Hi-C data.

Chromosome group	No. of contigs	Size of contigs (bp)
Chr1	2	48,438,208
Chr2	5	96,078,175
Chr3	3	59,867,354
Chr4	4	51,572,703
Chr5	2	49,605,055
Chr6	4	61,695,483
Chr7	2	48,766,362
Chr8	6	87,163,298
Chr9	7	52,076,336
Chr10	3	47,605,056
Chr11	8	74,520,300
Total	**46**	**677,388,330**

Supplementary Table 5. The genome assemblies analyzed in this study

Species	Genome size (bp)	Contig N50 (bp)	Scaffold N50 (bp)	Gene number	Source
Arachis duranensis	1,084,261,490	19,044	110,037,037	36,734	PeanutBase database
Arachis ipaensis	1,353,826,449	20,465	136,175,642	41,840	PeanutBase database
Arabidopsis thaliana	119,667,750	10,898,021	23,459,830	27,654	Phytozome database
Cicer arietinum	532,289,632	30,272	39,989,001	28,269	Phytozome database
Cajanus cajan	605,780,537	20,456	516,056	40,071	Phytozome database
Robinia pseudoacacia	**682,403,908**	**32,134,000**	**59,867,354**	**33,187**	**This study**
Glycine max	978,386,919	405,144	49,893,278	52,872	Phytozome database
Glycine soja	985,259,765	23,200	48,820,172	46,969	Phytozome database
Lupinus albus	450,972,408	8,734,267	17,349,171	38,258	Phytozome database
Lupinus angustifolius	609,204,147	46,346	21,299,880	33,072	Phytozome database
Lotus japonicus	544,173,911	2,515,607	85,550,444	28,251	Phytozome database
Medicago truncatula	411,831,487	218,749	49,172,423	50,894	Phytozome database
Phaseolus vulgaris	537,218,636	1,869,387	49,670,989	27,433	Phytozome database
Trifolium pratense	345,990,850	3,851	22,682,783	39,943	Phytozome database
Vigna radiata	463,085,359	22,539	25,360,630	22,368	Phytozome database
Vigna unguiculata	519,435,864	10,911,736	41,684,185	31,948	Phytozome database

Note: PeanutBase: https://peanutbase.org/home

Phytozome database: https://phytozome-next.jgi.doe.gov

Supplementary Table 6. BUSCO scores of the *R. pseudoacacia* genome assembly

Type	Number	Percent
Complete BUSCOs	1585	98.20%
Complete single-copy BUSCOs	1534	95.04%
Complete duplicated BUSCOs	51	3.16%
Fragmented BUSCOs	13	0.95%
Missing BUSCOs	16	0.99%
Total BUSCO groups searched	1614	

Supplementary Table 7. Genomic BUSCO scores of closely related legume species

Species	Type	Assembly Percent	Total BUSCO groups searched	Source article
Cicer arietinum	Complete BUSCOs	91.30%	1614	10.1016/j.jare.2021.10.009
	Complete single-copy BUSCOs	87.00%		
	Complete duplicated BUSCOs	4.30%		
	Fragmented BUSCOs	1.80%		
	Missing BUSCOs	6.90%		
Lotus japonicus	Complete BUSCOs	98.14%	1614	10.1093/dnares/dsaa015
	Complete single-copy BUSCOs	94.67%		
	Complete duplicated BUSCOs	3.47%		
	Fragmented BUSCOs	0.93%		
	Missing BUSCOs	0.93%		
Medicago sativa	Complete BUSCOs	93.30%	1440	10.1016/j.molp.2020.07.003
	Complete single-copy BUSCOs	87.80%		
	Complete duplicated BUSCOs	5.50%		
	Fragmented BUSCOs	1.00%		
	Missing BUSCOs	5.70%		
Cajanus cajan	Complete BUSCOs	90.40%	1614	10.1016/j.jare.2021.10.009
	Complete single-copy BUSCOs	84.40%		
	Complete duplicated BUSCOs	6.00%		
	Fragmented BUSCOs	2.80%		
	Missing BUSCOs	6.80%		
***Glycine max* Wm82v4**	Complete BUSCOs	93.20%	1440	10.1111/tpj.14500
	Complete single-copy BUSCOs	47.20%		
	Complete duplicated BUSCOs	46.00%		
	Fragmented BUSCOs	1.00%		
	Missing BUSCOs	5.80%		
***Glycine soja* PI 483463**	Complete BUSCOs	93.60%	1440	10.1111/tpj.14500
	Complete single-copy BUSCOs	46.90%		
	Complete duplicated BUSCOs	46.70%		
	Fragmented BUSCOs	0.90%		
	Missing BUSCOs	5.50%		

Supplementary Table 8. Classification of repetitive elements annotated in the *R. pseudoacacia* genome

Element type	Length occupied (bp)	Percentage of genome
DNA	45,066,254	6.60%
LTR	246,993,163	36.19%
LINE	19,399,530	2.84%
SINE	85,596	0.01%
Satellite	47,010	0.01%
Simple repeats	142,323	0.02%
other	3,067,075	0.45%
Unclassified	91,084,598	13.35%
Total	405,857,565	59.47%

Supplementary Table 9. Summary of protein-coding genes predicted in the *R. pseudoacacia* genome

Type	*R. pseudoacacia*
No. of protein-coding genes	33,187
Average Gene Length (bp)	4,492.45
Average CDS Length (bp)	1,223.56
Average Exons per Gene	5.16
Average Exon Length (bp)	237.27
Average Intron Length (bp)	649.12

Supplementary Table 10. Summary of functional annotation of protein-coding genes in the *R. pseudoacacia* genome

Database	No. of annotated genes	Percentage
GO	30,980	93.35%
KEGG	14,821	44.66%
InterPro	32,235	97.13%
Swiss-Prot	27,304	82.27%
TrEMBL	32,416	97.68%
NR	32,214	97.07%
Annotated	32,972	99.35%
All[a]	33,187	100.00%

[a] At least one match in any of the databases above.

Supplementary Table 11. BUSCO scores of the *R. pseudoacacia* gene set

Type	Number	Percent
Complete BUSCOs	1563	96.84%
Complete single-copy BUSCOs	1502	93.06%
Complete duplicated BUSCOs	61	3.78%
Fragmented BUSCOs	10	0.62%
Missing BUSCOs	41	2.54%
Total BUSCO groups searched	1614	

Supplementary Table 12. The selected enriched GO terms of the expanded gene families in *R. pseudoacacia*

ID	Description	Gene ratio	P value	Adjusted P value	Q value	Count
GO:0009595	detection of biotic stimulus	1.94	8.80E-32	6.41E-29	5.86E-29	229
GO:0039657	suppression by virus of host gene expression	2.87	2.07E-30	1.42E-27	1.29E-27	82
GO:0009821	alkaloid biosynthetic process	1.96	8.19E-27	3.44E-24	3.14E-24	186
GO:0016099	monoterpenoid biosynthetic process	2.26	5.42E-25	2.20E-22	2.01E-22	117
GO:0048544	recognition of pollen	1.87	3.90E-24	1.52E-21	1.39E-21	193
GO:0016098	monoterpenoid metabolic process	2.21	1.23E-23	4.19E-21	3.82E-21	117
GO:0051554	flavonol metabolic process	2.00	2.29E-21	6.39E-19	5.84E-19	138
GO:0016137	glycoside metabolic process	1.83	1.18E-20	2.92E-18	2.67E-18	175
GO:0052544	defense response by callose deposition in cell wall	1.82	5.89E-19	1.15E-16	1.05E-16	162
GO:0052482	defense response by cell wall thickening	1.82	5.89E-19	1.15E-16	1.05E-16	162
GO:0051553	flavone biosynthetic process	1.96	1.75E-18	3.19E-16	2.91E-16	126
GO:0052543	callose deposition in cell wall	1.71	2.56E-17	4.13E-15	3.77E-15	181
GO:0052386	cell wall thickening	1.68	4.91E-17	7.67E-15	7.00E-15	190
GO:0016045	detection of bacterium	1.85	1.03E-15	1.32E-13	1.20E-13	124
GO:0009807	lignan biosynthetic process	2.23	1.82E-15	2.22E-13	2.02E-13	72
GO:0009806	lignan metabolic process	2.23	1.82E-15	2.22E-13	2.02E-13	72
GO:0060320	rejection of self pollen	2.11	8.87E-15	9.33E-13	8.52E-13	80
GO:0009717	isoflavonoid biosynthetic process	2.10	3.73E-12	2.93E-10	2.68E-10	65
GO:0080027	response to herbivore	1.93	1.55E-11	1.11E-09	1.01E-09	77
GO:0033759	flavone synthase activity	2.10	2.25E-11	1.55E-09	1.41E-09	60
GO:0009992	cellular water homeostasis	2.18	7.00E-09	3.37E-07	3.08E-07	41
GO:1990641	response to iron ion starvation	1.73	8.77E-09	4.15E-07	3.79E-07	80
GO:0009403	toxin biosynthetic process	1.63	1.14E-08	5.33E-07	4.87E-07	99
GO:0010597	green leaf volatile biosynthetic process	2.19	4.21E-08	1.76E-06	1.61E-06	36
GO:0009625	response to insect	1.48	1.68E-07	6.29E-06	5.74E-06	126
GO:0010345	suberin biosynthetic process	1.50	5.33E-07	1.90E-05	1.73E-05	107
GO:0046274	lignin catabolic process	1.90	1.59E-06	5.06E-05	4.62E-05	41
GO:0043667	pollen wall	1.66	5.43E-06	1.44E-04	1.31E-04	58
GO:0009609	response to symbiotic bacterium	1.44	6.86E-06	1.78E-04	1.63E-04	106
GO:0009963	positive regulation of flavonoid biosynthetic process	1.47	9.75E-06	2.47E-04	2.25E-04	91
GO:0030104	water homeostasis	1.32	1.17E-05	2.93E-04	2.68E-04	168
GO:0045431	flavonol synthase activity	1.82	2.05E-05	4.87E-04	4.45E-04	37
GO:0044277	cell wall disassembly	1.66	2.23E-05	5.25E-04	4.79E-04	50
GO:2000067	regulation of root morphogenesis	1.38	4.88E-05	1.07E-03	9.77E-04	108
GO:0010022	meristem determinacy	1.44	7.92E-05	1.66E-03	1.52E-03	81
GO:0010030	positive regulation of seed germination	1.44	8.74E-05	1.78E-03	1.63E-03	79
GO:0042631	cellular response to water deprivation	1.31	1.18E-04	2.34E-03	2.13E-03	138
GO:0052325	cell wall pectin biosynthetic process	1.44	1.73E-04	3.14E-03	2.87E-03	72
GO:0046794	transport of virus	1.36	1.78E-04	3.22E-03	2.94E-03	103
GO:0090059	protoxylem development	1.67	1.89E-04	3.41E-03	3.11E-03	38
GO:0080092	regulation of pollen tube growth	1.33	2.02E-04	3.62E-03	3.31E-03	113

GO:0009962	regulation of flavonoid biosynthetic process	1.26	2.10E-04	3.75E-03	3.42E-03	168
GO:0048281	inflorescence morphogenesis	1.50	2.84E-04	4.82E-03	4.40E-03	56
GO:0010582	floral meristem determinacy	1.42	2.85E-04	4.83E-03	4.41E-03	73
GO:0071462	cellular response to water stimulus	1.29	2.87E-04	4.84E-03	4.41E-03	138
GO:0009819	drought recovery	1.54	4.96E-04	7.96E-03	7.27E-03	45
GO:0002213	defense response to insect	1.28	6.04E-04	9.54E-03	8.71E-03	131
GO:0010018	far-red light signaling pathway	1.47	6.55E-04	9.97E-03	9.10E-03	54
GO:0010451	floral meristem growth	1.57	6.72E-04	1.02E-02	9.32E-03	40
GO:1900376	regulation of secondary metabolite biosynthetic process	1.22	7.60E-04	1.13E-02	1.03E-02	183

Supplementary Table 13. Reference sequences of LAC gene family in *Arabidopsis thaliana*

**Gene symbol**	**Gene ID**
AtLAC1	AT1G18140
AtLAC2	AT2G29130
AtLAC3	AT2G30210
AtLAC4	AT2G38080
AtLAC5	AT2G40370
AtLAC6	AT2G46570
AtLAC7	AT3G09220
AtLAC8	AT5G01040
AtLAC9	AT5G01050
AtLAC10	AT5G01190
AtLAC11	AT5G03260
AtLAC12	AT5G05390
AtLAC13	AT5G07130
AtLAC14	AT5G09360
AtLAC15	AT5G48100
AtLAC16	AT5G58910
AtLAC17	AT5G60020
AtLAC15	AT5G48100
AtLAC16	AT5G58910
AtLAC17	AT5G60020

Supplementary Table 14. Summary of *R. pseudoacacia* individuals sampled in this study

**Populations**	**Location**	**Latitude**	**Longitude**	**Number of samples**
P1	Shuangcike, Minqin, Gansu Province, China	38.78	103.26	4
P2	Xuebai, Minqin, Gansu Province, China	38.56	103.00	5
P3	Lanzhouxinqu, Gansu Province, China	36.41	103.40	5
P4	Maoxian, Sichuan Province, China	31.77	103.79	5
P5	Lingbao, Henan Province, China	34.48	110.90	5
P6	Shennongjia, Hubei Province, China	31.51	110.39	5
P7	Xuanhan, Sichuan Province, China	31.38	107.71	5
P8	Foping, Shaanxi Province, China	33.38	108.02	5
P9	Liuba, Shaanxi Province, China	33.72	106.77	1
P10	Songpan, Sichuan Province, China	32.41	103.71	3
P11	Shennongjia, Hubei Province, China	31.79	110.53	5
P12	Huxian, Shaanxi Province, China	33.79	108.57	1
P13	Taibai, Shaanxi Province, China	33.82	107.62	5
P14	Zhouqu, Gansu Province, Chin	33.63	104.43	5
**Total**				**59**

Supplementary Table 15. Summary of whole genome re-sequencing data generated in this study

**Sample**	**Population**	**Reads Number**	**Bases Number (bp)**	**Mapping Ratio**	**Mapping Coverage**	**Depth (×)**
P1-1	P1	71,673,970	10,751,095,500	98.46%	91.05%	14.71
P1-2	P1	72,019,348	10,802,902,200	98.64%	90.75%	14.68
P1-3	P1	71,791,574	10,768,736,100	98.62%	90.84%	14.74
P1-4	P1	71,869,416	10,780,412,400	98.77%	90.73%	14.82
P2-1	P2	72,025,380	10,803,807,000	97.16%	90.05%	13.97
P2-2	P2	71,504,482	10,725,672,300	97.94%	90.80%	14.14
P2-3	P2	71,365,788	10,704,868,200	97.69%	90.76%	14.33
P2-4	P2	65,140,044	9,771,006,600	98.34%	90.67%	13.35
P2-5	P2	71,732,482	10,759,872,300	97.99%	90.78%	14.52
P3-1	P3	71,111,728	10,666,759,200	97.30%	90.64%	14.41
P3-2	P3	60,322,398	9,048,359,700	97.48%	90.64%	11.81
P3-3	P3	71,776,544	10,766,481,600	98.38%	90.54%	14.65
P3-4	P3	71,944,566	10,791,684,900	98.45%	90.51%	14.85
P3-5	P3	71,621,122	10,743,168,300	98.53%	90.77%	14.64
P4-1	P4	61,057,184	9,158,577,600	98.63%	90.79%	12.66
P4-2	P4	68,490,208	10,273,531,200	98.93%	90.90%	14.17
P4-3	P4	74,955,078	11,243,261,700	98.42%	90.97%	15.39
P4-4	P4	68,721,244	10,308,186,600	98.58%	90.76%	14.26
P4-5	P4	68,543,080	10,281,462,000	98.47%	90.66%	14.10
P5-1 *	P5	67,088,548	10,063,282,200	98.61%	90.95%	13.89
P5-2	P5	75,147,314	11,272,097,100	96.54%	90.81%	15.22
P5-3 *	P5	64,572,218	9,685,832,700	95.89%	90.76%	12.99

P5-4 *	P5	68,649,856	10,297,478,400	97.81%	90.98%	14.08
P5-5	P5	68,040,216	10,206,032,400	95.12%	90.25%	13.45
P6-1	P6	68,669,210	10,300,381,500	93.08%	90.78%	13.37
P6-2	P6	68,644,950	10,296,742,500	98.72%	90.83%	14.21
P6-3	P6	68,700,456	10,305,068,400	98.63%	90.56%	14.25
P6-4	P6	68,294,704	10,244,205,600	91.50%	90.63%	13.03
P6-5	P6	68,215,718	10,232,357,700	97.59%	90.39%	13.92
P7-1	P7	68,531,296	10,279,694,400	97.35%	90.65%	14.02
P7-2	P7	68,667,510	10,300,126,500	95.84%	90.52%	13.83
P7-3	P7	68,774,154	10,316,123,100	98.32%	90.88%	14.15
P7-4	P7	68,818,152	10,322,722,800	98.55%	90.81%	14.24
P7-5	P7	68,629,096	10,294,364,400	97.86%	90.73%	14.09
P8-1	P8	68,574,824	10,286,223,600	98.57%	90.76%	14.17
P8-2	P8	67,238,084	10,085,712,600	98.44%	90.70%	13.83
P8-3	P8	67,151,178	10,072,676,700	98.05%	90.75%	13.88
P8-4	P8	67,111,318	10,066,697,700	98.92%	90.72%	13.99
P8-5	P8	67,179,006	10,076,850,900	98.94%	90.90%	13.96
P9-1	P9	68,450,530	10,267,579,500	97.29%	90.73%	14.01
P10-1	P10	68,483,358	10,272,503,700	98.31%	90.41%	14.17
P10-2	P10	68,553,936	10,283,090,400	98.90%	90.70%	14.22
P10-3	P10	68,738,612	10,310,791,800	98.48%	90.77%	14.18
P11-1	P11	67,112,872	10,066,930,800	97.99%	90.66%	13.89
P11-2	P11	66,891,924	10,033,788,600	98.31%	90.51%	13.83
P11-3	P11	68,527,348	10,279,102,200	97.86%	90.93%	14.09
P11-4	P11	66,890,432	10,033,564,800	97.66%	90.74%	13.66
P11-5	P11	68,536,034	10,280,405,100	98.67%	90.61%	14.19
P12-1	P12	68,413,280	10,261,992,000	98.95%	90.34%	14.21
P13-1	P13	68,482,720	10,272,408,000	97.65%	90.56%	13.96
P13-2	P13	66,552,586	9,982,887,900	98.77%	90.80%	13.86
P13-3 **	P13	68,709,454	10,306,418,100	98.70%	91.01%	14.25
P13-4	P13	68,783,870	10,317,580,500	97.94%	90.55%	13.85
P13-5 **	P13	68,645,096	10,296,764,400	98.28%	90.39%	14.00
P14-1	P14	68,755,698	10,313,354,700	98.49%	90.88%	14.23
P14-2	P14	68,609,734	10,291,460,100	98.10%	90.58%	14.14
P14-3	P14	68,673,676	10,301,051,400	98.77%	90.71%	14.23
P14-4	P14	68,712,030	10,306,804,500	98.27%	90.69%	14.23
P14-5	P14	67,908,944	10,186,341,600	98.75%	91.20%	14.16
**Average**		**68,793,145**	**10,318,971,808**	**97.92%**	**90.71%**	**14.07**

Note: The two groups of outlier individuals are labeled with one or two asterisks (* or **), respectively.

Supplementary Table 16. Summary of π values of 29 plants

Species	π value	Source article
Glycine max (improved cultivars)	0.00105	10.1038/nbt.3096
Glycine max (landraces)	0.00140	10.1038/nbt.3096
Glycine soja (wild soybean)	0.00294	10.1038/nbt.3096
Phoenix dactylifera	0.00920	10.1038/ncomms9824
Betula pendula	0.00880	10.1038/ng.3862
Malus domestica	0.00220	10.1038/s41467-017-00336-7
Ostrya chinensis	0.00279	10.1038/s41467-018-07913-4
Ostrya rehderiana	0.00166	10.1038/s41467-018-07913-4
Ginkgo biloba	0.00257	10.1038/s41467-019-12133-5
Liriodendron chinense	0.00054	10.1038/s41477-018-0323-6
Phaseolus vulgaris (landraces)	0.00285	10.1038/s41588-019-0546-0
Phaseolus vulgaris (improved cultivars)	0.00302	10.1038/s41588-019-0546-0
Populus tremula	0.01470	10.1093/molbev/msw051
Populus tremuloides	0.01600	10.1093/molbev/msw051
Davidia involucrata	0.00585	10.1111/1755-0998.13138
Prunus persica (improved cultivars)	0.00100	10.1186/s13059-019-1648-9
Prunus persica (landraces)	0.00120	10.1186/s13059-019-1648-9
Prunus mira et al. (wild pea)	0.00350	10.1186/s13059-019-1648-9
Arabidopsis thaliana	0.00315	10.1371/journal.pbio.1002112
Brachypodium distachyon	0.00267	10.1371/journal.pbio.1002112
Capsella rubella	0.00327	10.1371/journal.pbio.1002112
Citrullus lanatus	0.00241	10.1371/journal.pbio.1002112
Citrus reticulata	0.01496	10.1371/journal.pbio.1002112
Cucumis sativus var. *hardwickii*	0.01321	10.1371/journal.pbio.1002112
Medicago truncatula	0.00514	10.1371/journal.pbio.1002112
Oryza rufipogon	0.00636	10.1371/journal.pbio.1002112
Populus trichocarpa	0.00317	10.1371/journal.pbio.1002112
Ziziphus jujuba	0.00219	10.1371/journal.pgen.1006433
Robinia pseudoacacia	**0.00894**	**This study**

Note: Landraces and cultivars are labeled in blue color. Wild lineages are labeled in black color. *Robinia pseudoacacia* is labeled in red color.

Supplementary Table 17. The enriched GO terms of the candidate PSGs in *R. pseudoacacia*

ID	Description	Gene ratio	P value	Adjusted P value	Q value	Count
GO:0034644	cellular response to UV	3.27	8.37E-07	2.48E-04	2.36E-04	23
GO:0016045	detection of bacterium	3.54	2.70E-05	6.47E-03	6.15E-03	15
GO:0052543	callose deposition in cell wall	2.68	1.65E-04	2.39E-02	2.27E-02	18
GO:0080110	sporopollenin biosynthetic process	4.58	1.61E-04	2.39E-02	2.27E-02	9
GO:0050829	defense response to Gram-negative bacterium	2.41	2.22E-04	3.02E-02	2.87E-02	21
GO:0010082	regulation of root meristem growth	2.47	2.27E-04	3.02E-02	2.87E-02	20
GO:0080027	response to herbivore	3.96	2.35E-04	3.04E-02	2.89E-02	10
GO:0052386	cell wall thickening	2.51	3.69E-04	4.27E-02	4.06E-02	18
GO:0016799	hydrolase activity, hydrolyzing N-glycosyl compounds	3.74	2.25E-10	2.42E-07	2.30E-07	32
GO:0003953	NAD+ nucleosidase activity	4.53	1.95E-10	2.42E-07	2.30E-07	26
GO:0050135	NAD(P)+ nucleosidase activity	4.53	1.95E-10	2.42E-07	2.30E-07	26
GO:0061809	NAD+ nucleotidase, cyclic ADP-ribose generating	4.53	1.95E-10	2.42E-07	2.30E-07	26
GO:0019364	pyridine nucleotide catabolic process	4.94	1.63E-10	2.42E-07	2.30E-07	24
GO:0019677	NAD catabolic process	4.94	1.63E-10	2.42E-07	2.30E-07	24
GO:0046496	nicotinamide nucleotide metabolic process	4.46	6.02E-10	5.57E-07	5.29E-07	25
GO:0019362	pyridine nucleotide metabolic process	4.41	7.46E-10	6.03E-07	5.73E-07	25
GO:0072526	pyridine-containing compound catabolic process	4.50	1.12E-09	8.06E-07	7.66E-07	24
GO:0009166	nucleotide catabolic process	3.96	3.47E-09	2.24E-06	2.13E-06	26
GO:1901292	nucleoside phosphate catabolic process	3.87	5.62E-09	3.31E-06	3.14E-06	26
GO:0072524	pyridine-containing compound metabolic process	3.83	6.71E-09	3.62E-06	3.44E-06	26
GO:0034404	nucleobase-containing small molecule biosynthetic process	3.47	8.70E-09	4.33E-06	4.12E-06	29
GO:0030275	LRR domain binding	4.47	2.97E-08	1.37E-05	1.30E-05	20
GO:0016849	phosphorus-oxygen lyase activity	3.43	6.25E-08	2.69E-05	2.56E-05	26
GO:0009975	cyclase activity	3.25	1.82E-07	7.35E-05	6.99E-05	26
GO:0009862	systemic acquired resistance, salicylic acid mediated signaling pathway	3.96	2.18E-07	8.31E-05	7.90E-05	20
GO:0004016	adenylate cyclase activity	4.02	3.43E-07	1.23E-04	1.17E-04	19
GO:0009870	defense response signaling pathway, resistance gene-dependent	4.28	5.95E-07	2.03E-04	1.93E-04	17
GO:0002758	innate immune response-activating signal transduction	2.93	8.44E-07	2.48E-04	2.36E-04	27
GO:0010193	response to ozone	3.18	8.10E-07	2.48E-04	2.36E-04	24
GO:0002230	positive regulation of defense response to virus by host	4.94	6.22E-06	1.75E-03	1.66E-03	12
GO:0050691	regulation of defense response to virus by host	4.42	1.96E-05	5.29E-03	5.02E-03	12
GO:0046500	S-adenosylmethionine metabolic process	5.84	2.37E-05	6.14E-03	5.83E-03	9
GO:0009595	detection of biotic stimulus	2.81	2.52E-05	6.26E-03	5.95E-03	21
GO:0098581	detection of external biotic stimulus	2.86	3.04E-05	7.02E-03	6.67E-03	20
GO:0034002	(R)-limonene synthase activity	18.87	4.05E-05	9.02E-03	8.58E-03	4
GO:0051278	fungal-type cell wall polysaccharide biosynthetic process	11.79	5.24E-05	1.13E-02	1.07E-02	5
GO:0098543	detection of other organism	3.33	5.49E-05	1.14E-02	1.09E-02	15
GO:0052482	defense response by cell wall thickening	3.01	6.26E-05	1.23E-02	1.17E-02	17
GO:0052544	defense response by callose deposition in cell wall	3.01	6.26E-05	1.23E-02	1.17E-02	17

GO:0070330	aromatase activity	6.85	6.81E-05	1.29E-02	1.23E-02	7
GO:0033383	geranyl diphosphate metabolic process	8.42	6.98E-05	1.29E-02	1.23E-02	6
GO:0102903	gamma-terpinene synthase activity	15.97	8.50E-05	1.53E-02	1.45E-02	4
GO:0045338	farnesyl diphosphate metabolic process	7.98	9.49E-05	1.66E-02	1.58E-02	6
GO:0045339	farnesyl diphosphate catabolic process	14.83	1.17E-04	1.89E-02	1.80E-02	4
GO:1901941	(+)-epi-alpha-bisabolol metabolic process	14.83	1.17E-04	1.89E-02	1.80E-02	4
GO:1901943	(+)-epi-alpha-bisabolol biosynthetic process	14.83	1.17E-04	1.89E-02	1.80E-02	4
GO:0002526	acute inflammatory response	7.41	1.45E-04	2.29E-02	2.17E-02	6
GO:0071966	fungal-type cell wall polysaccharide metabolic process	9.61	1.48E-04	2.29E-02	2.17E-02	5
GO:0033353	S-adenosylmethionine cycle	7.24	1.66E-04	2.39E-02	2.27E-02	6
GO:0030719	P granule organization	9.27	1.78E-04	2.50E-02	2.38E-02	5
GO:0016712	oxidoreductase activity, acting on paired donors, with incorporation or reduction of molecular oxygen, reduced flavin or flavoprotein as one donor, and incorporation of one atom of oxygen	5.68	2.29E-04	3.02E-02	2.87E-02	7
GO:0090219	negative regulation of lipid kinase activity	12.21	2.66E-04	3.37E-02	3.21E-02	4
GO:0006144	purine nucleobase metabolic process	4.25	2.85E-04	3.55E-02	3.37E-02	9
GO:0034008	R-linalool synthase activity	11.53	3.37E-04	3.96E-02	3.77E-02	4
GO:0044020	histone methyltransferase activity (H4-R3 specific)	11.53	3.37E-04	3.96E-02	3.77E-02	4
GO:0070880	fungal-type cell wall beta-glucan biosynthetic process	11.53	3.37E-04	3.96E-02	3.77E-02	4
GO:0031904	endosome lumen	10.92	4.20E-04	4.69E-02	4.46E-02	4
GO:0043985	histone H4-R3 methylation	10.92	4.20E-04	4.69E-02	4.46E-02	4
GO:0099568	cytoplasmic region	2.46	4.60E-04	5.05E-02	4.80E-02	18
