# Supplementary material for: Chromosome-level genome assembly and population genomics of Robinia pseudoacacia reveal the genetic basis for its wide cultivation
Source: Commun Biol. 2023 Jul 31;6:797. doi: 10.1038/s42003-023-05158-6 (PMC10390555; doi:10.1038/s42003-023-05158-6)
Supplement: Supplementary file 3 — Description of Additional Supplementary Files [file 42003_2023_5158_MOESM3_ESM.pdf]

Description of Additional Supplementary Files

File name: Supplementary Data 1
Description: Gene list of the selected enriched GO terms of the expanded gene families involved in wood formation in *R. pseudoacacia*.

File name: Supplementary Data 2
Description: The positively selected genes identified in *R. pseudoacacia*.

File name: Supplementary Data 3
Description: *K*s peaks distribution in *R. pseudoacacia*, *G. max* and *M. truncatula*.
